# Synthesis of *bis*-Chalcones Based on Green Chemistry Strategies and Their Cytotoxicity Toward Human MeWo and A375 Melanoma Cell Lines

**DOI:** 10.3390/molecules29215171

**Published:** 2024-10-31

**Authors:** Dorota Olender, Anna Pawełczyk, Anna Leśków, Katarzyna Sowa-Kasprzak, Lucjusz Zaprutko, Dorota Diakowska

**Affiliations:** 1 Chair and Department of Organic Chemistry, Faculty of Pharmacy, Poznan University of Medical Sciences, Rokietnicka 3, 60-806 Poznań, Poland; apaw@ump.edu.pl (A.P.); kasik@ump.edu.pl (K.S.-K.); zaprutko@ump.edu.pl (L.Z.); 2 Department of Medical Biology, Wroclaw Medical University, Chalubinskiego 3, 50-368 Wrocław, Poland; anna.leskow@umw.edu.pl (A.L.); dorota.diakowska@umw.edu.pl (D.D.)

**Keywords:** *bis*-chalcones, Claisen–Schmidt reaction, green chemistry, microwave- and ultrasound-assisted synthesis, cytotoxicity, anticancer activity

## Abstract

Chalcone is an aromatic ketone that forms the central core of many important biological compounds. Chalcone derivatives show various biological activities, especially anti-inflammatory, antibacterial, antioxidant, and anticancer activities, and also inhibit melanoma cell growth. In this study, we synthesized chalcone compounds with *bis*-chalcone’s chemical structure under microwave (MW) and microwave–ultrasound (MW-US) conditions and compared them to chalcones produced using the classical synthesis method. All *bis*-chalcones were synthesized with terephthalaldehyde and an appropriate aromatic ketone as substrates in Claisen–Schmidt condensation. All the obtained compounds were tested regarding their roles as potential anticancer agents. The cytotoxic effect of the *bis*-chalcones against human MeWo and A375 melanoma cell lines was investigated through colorimetric MTT and SRB assays. The data were analyzed statistically. In the case of the synthesis of *bis*-chalcones, it was determined that the use of green conditions supported by the MW or MW-US factors led to an increase in the yield of the final products and a reduction in the reaction time compared to the classic method. The biological results showed the high cytotoxic effect of *bis*-chalcones. The present results show the compounds’ high antiproliferative and cytotoxic potential, especially for the two selected *bis*-chalcone derivatives (**3b** and **3c**), in particular, at concentrations of 50 μM–200 μM at 24, 48 h, and 72 h of incubation. The use of MW and US for the synthesis of *bis*-chalcones significantly improved the process compared to the classical method. The derivatives containing two hydroxy and two methoxy groups were the most effective against the tested cancer cells.

## 1. Introduction

Current research on multifactorial diseases such as cancer and cardiovascular and neurodegenerative diseases reveals that designing new structures that determine multi-target biological activity is based on small-molecule frameworks with an effective pharmacology. Moreover, to increase and/or modify biological activity, it is advantageous to use pleiotropic compounds of natural origin or to introduce simple heterocyclic systems.

Cancer is still a life-threatening disease and the leading cause of death worldwide [1]. The determinants of cancer are, to a large extent, inflammation and the activation of the immune system [2]. While cancer is a multifactorial disease with many determinants, chronic inflammation and dysregulated immune responses are recognized as key factors in its initiation, progression, and metastasis. Therefore, new drugs and treatment methods are being sought that can be used to treat primary lesions that are resistant to existing methods, as this will increase the effectiveness of the current treatments and, at the same time, have fewer side effects.

*Melanoma malignum,* a kind of skin cancer, typically starts on skin that is often exposed to ultraviolet (UV) radiation, especially UVB, from sunlight and artificial sources (e.g., solaria). This includes the skin on the arms, back, face, and legs, but sometimes melanoma can form in the eyes or, rarely, inside the body. Scientists also point out that the most prevalent malignancy in humans is also a result of specific genetic susceptibility [3]. In the last decade, there have been significant breakthroughs in the treatment of advanced melanomas. More promising drugs and different immunotherapies have been applied [3,4]. These drugs have significantly increased the progression-free and overall survival for many patients with melanoma and also, most importantly, have lowered the mortality for this disease compared to a few years ago [3]. Today, numerous ongoing trials continue to evaluate the roles of novel therapies and new drugs for this challenging disease. It turns out that many natural compounds have shown promising anti-melanoma activity. Data from the literature suggest that naturally derived compounds, including flavonoids, triterpenoids, and chalcones, may be promising candidate compounds for treating different types of melanoma [5,6,7,8,9].

Chalcones are one of the numerous groups of natural compounds widely found in the plant world. They exhibit various biological activities, especially anti-inflammatory, antibacterial, antioxidant, and anticancer activities [10,11,12]. Chalcone derivatives have been the subject of many studies, and they also inhibit melanoma cell growth [13]. The skeletal structure of a chalcone is relatively simple but allows for chemical modifications and the subsequent investigation of antitumor treatments using the resulting new chalcone derivatives. Chemical strategies include the structural manipulation of two aryl rings, the substitution of aryl rings to generate heteroaryl scaffolds, and/or molecular hybridization through conjugation with other pharmacologically attractive scaffolds to enhance molecular anticancer properties [14]. It has been established that the presence of at least one phenyl ring with a hydroxy, methoxy, or acetyl group in the tested molecule is necessary for the occurrence of anticancer activity [15]. Moreover, a heterocyclic system in chalcone derivatives, specifically those incorporating five or more heteroatoms (N, S) in their structures, plays a role in anticancer activity [16]. Furthermore, combining the chalcone structure with other biologically active units is advantageous. Anticancer activity is presented by chalcone hybrids with the following structures: artemisinin–chalcone; chalcone–azole; chalcone–coumarin; and chalcone–indole types [17,18]. Our earlier biological studies also showed that curcuminoid chalcones, compounds that bear structural elements characteristic of curcumin and chalcone skeletons, have promising anticancer potential [19,20]. A chalcone with hydroxy and methoxy groups in aryl rings reduced the expression of antioxidant enzymes that could degrade ROS (reactive oxygen species), which is good news for multifactor diseases. The results also indicated that derivatives of this type exhibited significant toxicity toward the Caco-2 cell line [19]. Moreover, biological tests allowed for a preliminary assessment of the cytotoxic effect of synthetic curcuminoid chalcone hybrids with NSAIDs (non-steroidal anti-inflammatory drugs) in combination with ultrasound in head and neck cancer cell lines (SCC-25 and FaDu) [20]. Some of the tested curcuminoid chalcones may be effective sonosensitizers in sonodynamic therapy. Moreover, *bis*-chalcone compounds containing two chalcone units in one molecule are known in the literature and show different biological activities, e.g., anticancer [21,22,23]. Numerous studies have indicated that *bis*-chalcones inhibit the growth of human breast and colon cancer cell growth in cultures [24,25]. *Bis*-chalcones based on furan derivatives linked to aliphatic linkers showed more significant anticancer activity against lung and skin cancer cell lines compared with that of known chemotherapeutic drugs (doxorubicin) [26]. *Bis*-chalcones containing methoxyl or fluorine also demonstrated more potent cytotoxicity than curcumin and other *bis*-chalcone analogs tested [27]. Moreover, another study revealed that *bis*-chalcones consisting of substituted 4-hydroxy chalcones connected via an ether linkage with three-carbon linkages were characterized by more significant inhibition than the simple 4-hydroxy chalcone series.

In these studies, the corresponding *bis*-chalcones for melanoma treatment were obtained under the alternative microwave (MW)- and microwave–ultrasound (MW-US)-assisted synthesis conditions. MW and US are powerful drivers of chemical reactions and offer several advantages over classical conditions in terms of reaction efficiency, selectivity, and sustainability. Therefore, these techniques are widely used for the synthesis of different compounds, including chalcones and their derivatives [19,28].

## 2. Results and Discussion

### 2.1. Synthesis and Characterization of bis-Chalcone Derivatives Under MW and US Conditions

**Scheme 1:**
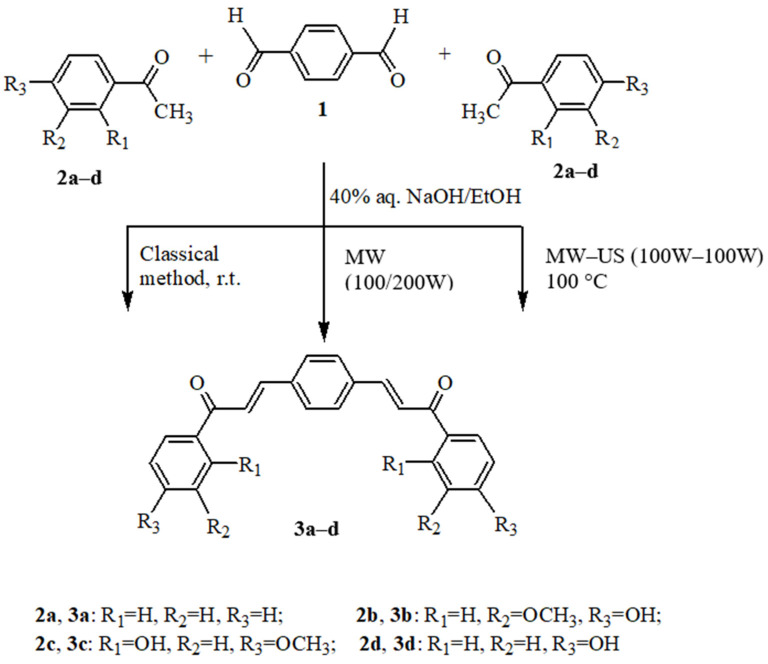
Synthesis of *bis*-chalcones **3a**–**3d** under the classical method, alternative microwave, and microwave–ultrasound-assisted synthesis conditions.

A series of four *bis*-chalcones (**3a**–**3d**) were synthesized via microwave- and ultrasound-assisted green synthesis in one-pot reactions using readily available starting materials, i.e., aromatic dialdehyde–terephthalaldehyde (**1**) and aromatic methylketones: acetophenone (**2a**); 4-hydroxy-3-methoxy-acetophenone (apocynin) (**2b**); 2-hydroxy-4-methoxy-acetophenone (paeonol) (**2c**); and 4-hydroxy-acetophenone (**2d**) (Scheme 1).

Difunctional terephtalaldehyde (**1**) reacted with appropriate carbanion formed in an alkaline medium from aromatic ketone, including acetophenone (**2a**), apocynin (**2b**), paeonol (**2c**), and piceol (**2d**), generating β-hydroxy carbonyl intermediates. This intermediate spontaneously produced *bis*-chalcone under the acidic condition. The selected acetophenones and terephthalaldehyde formed the corresponding *bis*-chalcones in the Claisen–Schmidt reaction using the procedure known from the literature data [19] for curcuminoid chalcones with modifications. In all the obtained products, terephthalaldehyde was condensed with twice the quantities of the appropriated acetophenone to yield the final *bis*-chalcone system. In the classic method, the reactions were carried out in an ethanolic solution of aromatic ketone, to which sodium hydroxide aqueous solution (40%, NaOH) and then an ethanolic solution of terephthalaldehyde (**1**) were added at room temperature. These reactions proceeded with good yields (79–88%); however, it took a relatively long time of 48 h [29]. As a result, four *bis*-chalcones (**3a**–**3d**) were obtained (Scheme 1), and the spectral characteristics were consistent with our recent studies [29].

Claisen–Schmidt condensation is a widely used method for the synthesis of different chalcone systems, including *bis*-chalcones. There are many reports on synthesizing chalcones via conventional methods [21]. However, these methods are not without limitations, with the primary disadvantages being their slow reaction rates, often lower yields, and formation of by-products [30]. Therefore, due to the long reaction time required for the complete synthesis of *bis*-chalcone compounds, it was decided to use factors supporting the chemical process, such as MW and US.

Microwave heating has been established as an incontestable method of organic reaction activation in laboratory practices, especially for its efficacy and environmentally friendly nature. MW irradiation provides rapid and uniform heating, significantly reducing the reaction times compared to conventional heating methods. It can also be more energy efficient, as it directly couples with the reactants, minimizing the energy loss to the environment. The increased temperature and energy absorption by polar molecules often lead to higher reaction rates and improved yields. MW can selectively heat specific components in a reaction mixture, leading to enhanced selectivity and reduced side reactions. The rate enhancements in these organic reactions can be attributed to the rapid superheating of the solvent, which facilitates faster reaction kinetics.

US, similar to MW irradiation, is also consistent with the principles of green chemistry. US induces cavitation, generating localized hot spots with extremely high temperatures and pressures, enhancing the reaction rates. The mechanical effects of US waves improve the mass transfer, facilitating the diffusion of reactants to the reaction sites. Reactions can often be performed under milder conditions (e.g., lower temperatures) compared to traditional methods.

The Claisen–Schmidt reaction is well-suited for MW- and US-assisted synthesis, and it can be performed in significantly less time than traditional methods while still providing good yields of the desired products. Although the mechanisms of cavitation and microwave effects are not fully understood, these green techniques will benefit the processes requiring enhanced heat transfer and mass transport [31]. Our previous work [19] presented favorable research results on the use of MW irradiation for the synthesis of curcuminoid chalcones. It has been shown that MW-assisted reactions can achieve favorable reaction parameters for the solvent-free microwave procedure. Processes conducted in the presence of US did not produce favorable results under the conditions used. Therefore, it was also decided to green the reaction for obtaining *bis*-chalcones. In these synthetic studies, the microwave procedure was used for the reaction in a polar solvent (alkaline water–alcoholic solution), similar to the classical method. In this case, it will be easy to determine the influence of the MW irradiation on the reduction in the reaction time of the synthesis of *bis*-chalcones (**3a**–**3d**). Several experiments were designed in microwave-only conditions (MW at 100 W and 200 W) and cross-combination conditions of MW and US factors (MW at 100 W and US at 100 W).

Under MW conditions, there was a significant reduction in the reaction time of *bis*-chalcone synthesis. The time was reduced from 48 h to 15 min at 100 W microwaves. An increase in the microwave power from 100 W to 200 W further shortened the reaction time to 10 min. However, the reaction occurred faster in the synergistic MW-US system, where the reaction time was reduced to 5 min. It should be noted that in the case of MW-assisted reactions, the reaction temperature increased as a result of the action of the MW irradiation. Finally, the MW- and MW-US-assisted processes were carried out at 80–85 °C (Table 1).

### 2.2. Biological Studies

The obtained *bis*-chalcones were examined for their potency as anticancer agents in a biological study.

#### 2.2.1. The Anticancer Activity of *bis*-Chalcone Derivatives on Human MeWo and A375 Melanoma Cell Lines

The cytotoxicity screening aimed to identify an active moiety that could become a successful drug in future generations. The synthesized compounds were subjected to an in vitro cytotoxicity assay against human MeWo and A375 melanoma cell lines with 1,3-diphenylprop-2-en-1-one (**ChO**) as a reference substance. The effects of the selected chalcone and *bis*-chalcones concentrations on the viability of the cells were evaluated following a treatment period of 24 h, 48 h, and 72 h using 3-(4,5-dimethylthiazol-2-yl)-2,5-diphenyl-2H-tetrazolium bromide (MTT) and the sulforhodamine B (SRB) assays, which have been widely used to conduct screening assays to investigate cytotoxicity in the cell-based studies.

##### The Cytotoxic Activity of *bis*-Chalcone Derivatives on the Viability of the Human MeWo Cell Line

**Figure 1:**
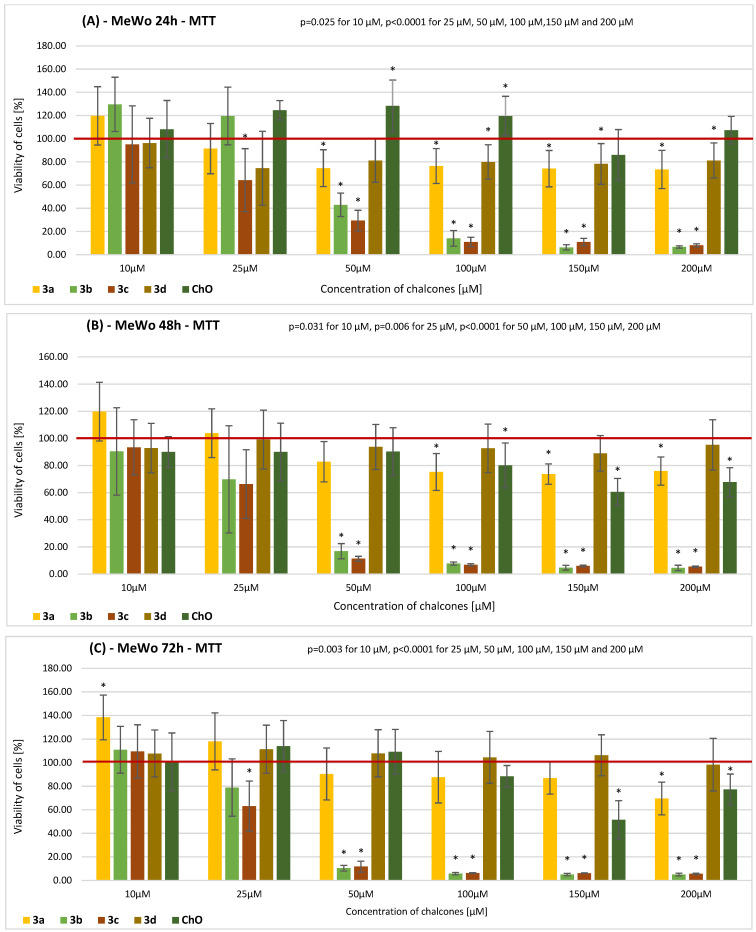
The results of the MTT assay on the viability of MeWo cells incubated with *bis*-chalcones **3a**–**3d** and **ChO** for (**A**) 24 h, (**B**) 48 h, and (**C**) 72 h. Descriptive data are presented as the mean ± SD. The results of the ANOVA analysis are described in figures (**A**–**C**). Statistically significant differences versus the control cells are marked with an asterisk (* *p* ≤ 0.0001). The horizontal bold line represents the viability of the control (0.2% DMSO-treated) cell.

All derivatives at a concentration above 25 µM exerted cytotoxic activity after 24 h of incubation (Table S1 in the Supplementary Materials). Two derivatives, **3b** and **3c**, had the best activity. The compound **3b** at 150 µM reduced the MeWo cell viability to 6.34%, and the **3c** at 200 µM reduced the MeWo cell viability to 8.14% (Figure 1A). After 48 h, the same derivatives at the highest tested concentration of 200 µM significantly decreased the cell viability to 4.54 and 5.42%, respectively. The cell viability at 150 µM was similar, with 4.69 and 5.42%, respectively (Figure 1B). After 72 h of treatment with *bis*-chalcones, the MeWo melanoma cell viability was similarly reduced by compounds **3b** and **3c**. The cell viability was significantly decreased at the following concentrations: 10.47% for compound **3b** at 50 µM and 5.76% for compound **3b** at 100 µM (Figure 1C). We did not observe significant cytotoxic effects for the remaining tested derivatives **3a** and **3d** after 24, 48, and 72 h of incubation (Figure 1A–C). There was also no statistically significant reduction in cell survival after **ChO** treatment (Table S1 in the Supplementary Materials). Since a statistically significant cytotoxic effect of derivatives **3b** and **3c** was already observed after 24 h of incubation, the IC_50_ values were calculated for the data from these measurements. For the MeWo cell line, the IC_50_ for derivative **3b** was 47.71 µM, and the IC_50_ for derivative **3c** was 35.26 µM.

**Figure 2:**
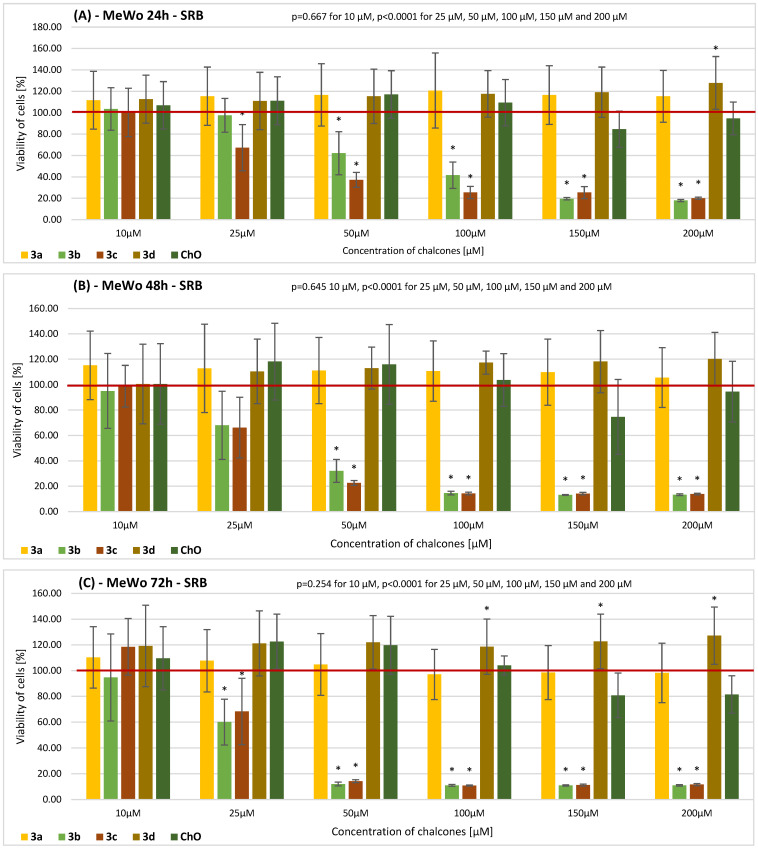
The results of the SRB assay on the viability of MeWo cells incubated with chalcones **3a**–**3d** and **ChO** for (**A**) 24 h, (**B**) 48 h, and (**C**) 72 h. Descriptive data are presented as the mean ± SD. The results of the ANOVA analysis are described in figures (**A**–**C**). Statistically significant differences versus the control cells are marked with an asterisk (* *p* ≤ 0.0001). The horizontal bold line represents the viability of the control (0.2% DMSO-treated) cells.

The SRB test is used to assess the number of proteins in living cells, and on this basis, we assessed the toxic effect of the tested substances on the MeWo cells (Table S2 in the Supplementary Materials). As shown in Figure 2, the results indicate that the tested compounds did not have a significant toxic effect on the MeWo cells and did not cause a statistically significant reduction in the number of viable cells, regardless of the tested concentration of these compounds or the time of incubation. Both compounds **3b** and **3c** exhibited higher cytotoxicity effects than the remaining compounds. The cell viability was significantly decreased to 10.85% at 100 µM for **3c** after 72 h of treatment (Figure 2C and Table S2 in the Supplementary Materials). For this test, the IC _50_ values were as follows: for derivative **3b**, −79.71 µM, and compound **3c**, −39.44 µM.

##### The Cytotoxic Activity of *bis*-Chalcone Derivatives on the Viability of A375 Cell Line

**Figure 3:**
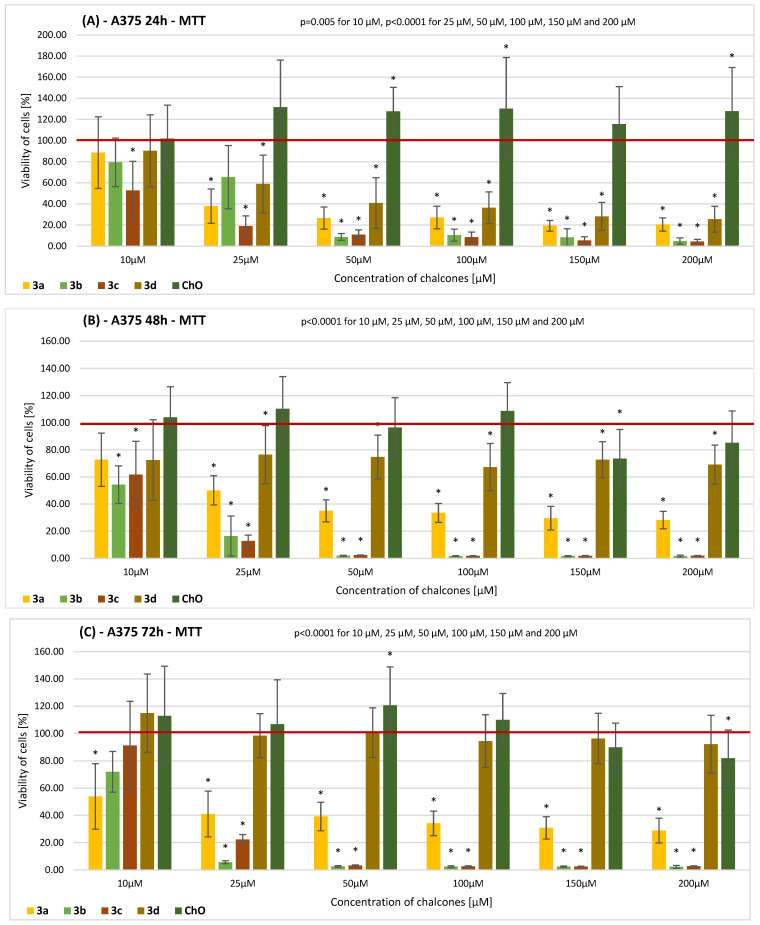
The results of the MTT assay on the viability of A375 cells incubated with chalcones **3a**–**3d** and **ChO** for (**A**) 24 h, (**B**) 48 h, and (**C**) 72 h. Descriptive data are presented as the mean ± SD. The results of the ANOVA analysis are described in figures (**A**–**C**). Statistically significant differences versus the control cells are marked with an asterisk (* *p* ≤ 0.0001). The horizontal bold line represents the viability of the control (0.2% DMSO-treated) cells.

**Figure 4:**
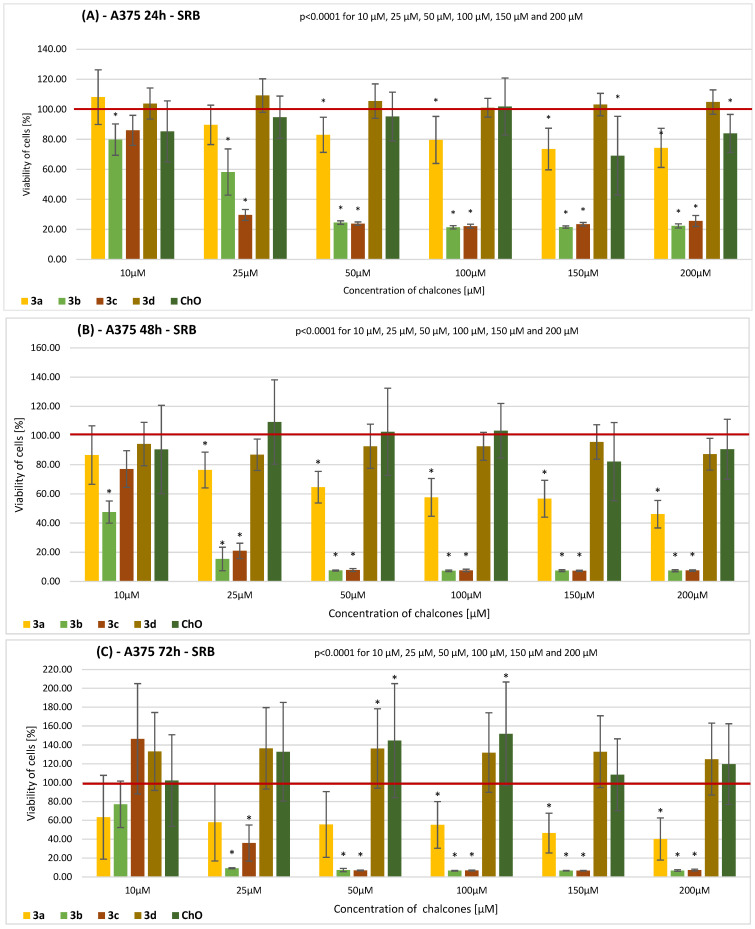
The results of the SRB assay on the viability of A375 cells incubated with chalcones **3a**–**3d** and **ChO** for (**A**) 24 h, (**B**) 48 h, and (**C**) 72 h. Descriptive data are presented as the mean ± SD. The results of the ANOVA analysis are described in figures (**A**–**C**). Statistically significant differences versus the control cells are marked with an asterisk (* *p* ≤ 0.0001). The horizontal bold line represents the viability of the control (0.2% DMSO-treated) cells.

The effects of selected chalcone and *bis*-chalcones concentrations on the viability of the A375 cells were evaluated following a treatment period of 24 h, 48 h, and 72 h and also using an MTT and SRB assay (Figure 3 and Figure 4 and Tables S3 and S4 in the Supplementary Materials).

All the derivatives showed cytotoxic activity on the viability of the A375 cells after 24 h. The best-decreased cell viability was observed for compounds **3b** and **3c** at 50–200 µM. The value of the IC_50_ for **3b** was 31.78 µM, and the IC_50_ for **3c** was 9.49 µM. The compound **3a** at 25 µM was relatively active. In other concentrations, the cell viability of A-375 was at a similar level and amounted to 20–26%. Similarly, increased activity was observed in the case of compound **3d**. With the dilution in the concentrations of compound **3d**, a lower cell viability rate was observed at the 90–25% level (Figure 3A and Table S3 in the Supplementary Materials).

After 48 h, a significantly higher cell viability was observed for compound **3d** because the cell viability was 72–68%. The best cytotoxic activity of compounds **3b** and **3c** was observed, and the longer incubation time involved decreased cell viability, especially at 50–200 µM. After 48 h, the cell viability decreased from 65% (for 24 h) to 15% for **3b** at 25 µM (Figure 3B and Table S3 in the Supplementary Materials).

After 72 h of treatment with *bis*-chalcones **3b** and **3c**, in concentrations above and equal to 50 µM, the A-375 cell viability remained at a low level of 5–6% (Figure 3C and Table S3 in the Supplementary Materials).

The results showed that the tested compounds were less cytotoxic than the A375 cells when the cytotoxic activity was evaluated using an SRB assay (Figure 4 and Table S4 in the Supplementary Materials). As with the MTT method, compounds **3b** and **3c** were the most active. After 48 h, a similar cell viability value was obtained compared to the MTT assay. The IC_50_ value for derivative **3b** was 31.11 µM, and for **3c**, it was 19.58 µM. A significant decrease in the cell viability was observed in the case of compound **3b** at a concentration of 25 µM, from 58% to 15%, respectively (Figure 4B). After 72 h of treatment with compounds **3b** and **3c**, a further decrease in the A-375 cell viability was noted. The same derivatives at the highest tested concentration of 200 µM significantly decreased the cell viability to 6.68 and 7.20%, respectively (Figure 4C). Moreover, the A-375 cell viability was significantly reduced by **3a** at 10 and 25 µM to 63.36 and 58.02%, respectively.

## 3. Materials and Methods

### 3.1. Solvents and Chemicals

A 40% aqueous sodium hydroxide solution (NaOH), terephthalaldehyde, acetophenone, 4-hydroxy-3-methoxyacetophenone (apocynin), 2-hydroxy-4-methoxyacetophenon, 4-hydroxyacetophenone (piceol), and solvents (ethanol, methanol, dichloromethane, hexane, ethyl acetate) from Aldrich (Saint Louis, MO, USA), Fluka (Buchs, Switzerland), Chempur (Piekary Śląskie, Poland), and POCh S.A. (Gliwice, Poland) were used.

All other chemicals of the highest purity were commercially available, and demineralized water was used in the tests.

### 3.2. Instrumental Analysis

The structures of compounds **3a**–**3d**, which were synthesized under nonclassical conditions, were confirmed by measuring the melting point and using spectral methods. The obtained results agreed with those published earlier [29]. The melting points were determined on a Boetius apparatus and were uncorrected. The IR spectra were recorded using a Nicolet iS50 FT-IR spectrometer (Thermo Scientific, Waltham, MA, USA). The ^1^H and ^13^C NMR spectra were recorded using an NMR Varian VNMR-S 400 MHz spectrometer at 400 and 100 MHz, respectively (Agilent Technologies, Santa Clara, CA, USA). The chemical shifts were expressed in parts per million (ppm) relative to tetramethylsilane (TMS) as an internal standard, using DMSO-*d*_6_ as the solvent. The MS spectra were recorded on a Bruker 320MS/420GC spectrometer apparatus (Bruker Corporation, Billerica, MA, USA) using the electron impact technique (EI), operating at 75 eV. The progress of the reactions and the purity of the products were checked using the TLC method on silica gel plates (DC-Alufolien Kieselgel 60 F_254_ from Merck, Darmstadt, Germany). Hexane and ethyl acetate (2:1, 4:3, 9:1, *v*/*v*) or chloroform and methanol (9:1, *v*/*v*) were used as the eluents. The TLC spots on the plates were observed in UV light (λ = 254 nm). Silica gel 60 (63–200 μm particle size, Merck) was used for the column chromatography. The crude reaction products were purified using a crystallization process or flash column chromatography using hexane and ethyl acetate (2:1, *v*/*v*).

### 3.3. Microwave- and Microwave–Ultrasound-Assisted Synthesis

MW and MW-US reactions were carried out in a UWave-1000 reactor (Sineo Microwave Chemistry Technology, Shanghai, China) equipped with a microwave generator (0–1000 W), an ultrasonic probe (26–28 kHz, 0–800 W), a UV lamp (λ = 365 nm, 300 W), a contact and non-contact thermometer, a magnetic stirrer, a cooler, and a reaction visualization system.

### 3.4. General Procedures of bis-Chalcone Synthesis

#### 3.4.1. Classic Method

An aqueous solution of 40% NaOH (5 mL) was added dropwise into a solution of 6 mmol of aromatic methylketone (acetophenone, apocynin, paeonol, piceol) in ethanol (20 mL). A total of 3 mmol of terephthalaldehyde in an ethanolic solution (5 mL) was introduced into the mixture and stirred at room temperature for 48 h. The reaction mixture was then poured into ice water and neutralized with 10% HCl to produce precipitates. The solid precipitates were filtered, washed with water, and crystallized with methanol to yield the final compounds. The aquatic solution was extracted with dichloromethane when the solid was not formed. The combined organic layer was washed with 10% HCl and then with water. The organic layer was dried over anhydrous MgSO_4_, and the solvent was removed under reduced pressure. The crude solid was purified via column chromatography using chloroform:methanol (9:1, *v*/*v*) as an eluent.

#### 3.4.2. Microwave Method

An aqueous solution of 40% NaOH (5 mL) was added dropwise into a solution of 6 mmol of aromatic methylketone (acetophenone, apocynin, paeonol, piceol) in ethanol (20 mL). A total of 3 mmol of terephthalaldehyde in an ethanolic solution (5 mL) was introduced into the mixture and was exposed to MW (100 W) for 15 min and 200 W for 10 min at 80–85 °C (reflux). The mixture was then poured into ice water and neutralized with 10% HCl to produce precipitates. The solid precipitates were filtered, washed with water, and crystallized with methanol to yield the final compounds. The aquatic solution was extracted with dichloromethane when the solid was not formed. The combined organic layer was washed with 10% HCl and then with water. The organic layer was dried over anhydrous MgSO_4_, and the solvent was removed under reduced pressure. The crude solid was purified via column chromatography using chloroform:methanol (9:1, *v*/*v*) as an eluent.

#### 3.4.3. Microwave–Ultrasound Method

An aqueous solution of 40% NaOH (5 mL) was added dropwise into a solution of 6 mmol of aromatic methylketone (acetophenone, apocynin, paeonol, piceol) in ethanol (20 mL). A total of 3 mmol of terephthalaldehyde in an ethanolic solution (5 mL) was introduced into the mixture and was simultaneously exposed to MW (100 W) and US (100 W) for 5 min at 80–85 °C (reflux). The mixture was then poured into ice water and neutralized with 10% HCl to produce precipitates. The solid precipitates were filtered, washed with water, and crystallized with methanol to yield the final compounds. The aquatic solution was extracted with dichloromethane when the solid was not formed. The combined organic layer was washed with 10% HCl and then with water. The organic layer was dried over anhydrous MgSO_4_, and the solvent was removed under reduced pressure. The crude solid was purified via column chromatography using chloroform:methanol (9:1, *v*/*v*) as an eluent.

The identity of the obtained compounds (**3a**–**3d**) was in agreement with the spectral data characteristics for products received in the classical reactions [29].

### 3.5. The Effect of the bis-Chalcone Derivatives on Human MeWo and A375 Melanoma Cell Lines

#### 3.5.1. Cell Culture and Application of the Tested Compounds

Human malignant melanoma cell lines A375 and MeWo (ATCC^®^ CRL-1619™ and HTB-65™ respectively; ATCC; Manassas, VA, USA) were cultured in culture flasks (T-75, Falcon^®^, Corning Life Sciences, Tewksbury, MA, USA) in a complete growth medium consisting of Dulbecco’s Modified Eagle Medium without phenol red (DMEM; Gibco, Thermo Fisher Scientific, Waltham, MA, USA), supplemented with 10% heat-inactivated fetal bovine serum (FBS; Gibco, Thermo Fisher Scientific, Waltham, MA, USA), with the addition of the stabilized 1% antibiotic antimycotic solution containing 25 µg/mL of amphotericin B, 10,000 units/mL of penicillin, and 10 mg/mL of streptomycin (Sigma-Aldrich, St. Luis, MO, USA). Cells were cultured in a NU-5710E In-vitroCell ES CO_2_ incubator (NuAire, Plymouth, MN, USA) at 37 °C in 95% humidified air with 5% CO_2_. The medium was renewed every three days. Cells were harvested from cell culture T-75 flasks (Eppendorf AG, Hamburg, Germany) with TrypLE™ Express (Gibco, Thermo Fisher Scientific, Waltham, MA, USA), stained with 0.4% trypan blue solution, and counted with a Countess™ Automated Cell Counter (Invitrogen, Thermo Fisher Scientific, Waltham, MA, USA). For the experiments, the cells were harvested with TrypLE™ Express (Gibco, Thermo Fisher Scientific, Waltham, MA, USA), then stained with 0.4% trypan blue solution, and counted with the use of a Countess™ Automated Cell Counter (Invitrogen, Thermo Fisher Scientific, Waltham, MA, USA). The cells were seeded at 1.2 × 10^4^ cells per well on 96-well tissue culture-treated microplates (Thermo Fisher Scientific, Waltham, MA, USA) and incubated overnight to allow attachment. The next day, the medium was removed, replaced with 100 µL of the tested compounds’ solution at 10–200 μM concentration, and incubated for 24, 48, and 72 h. Control cells were incubated in a complete growth medium with 0.2% dimethyl sulfoxide (DMSO) solvent (Invitrogen, Thermo Fisher Scientific, Waltham, MA, USA). In addition, non-treated controls, incubated in the pure complete growth medium, were created. This experiment was performed in triplicate, utilizing cells from different cell passages. Each series consisted of 3 replicates corresponding to different growth conditions (variable concentrations and type of tested compound).

#### 3.5.2. Evaluation of the Cell Metabolic Activity of Cells Treated with *bis*-Chalcone Derivatives with the MTT Assay

After the treatment time specified in the experiment conditions, the post-culture medium was removed gently, the cells were rinsed with sterile phosphate-buffered saline (PBS; Gibco, Thermo Fisher Scientific, Waltham, MA, USA) solution, and a freshly prepared 0.5 mg/mL 3-(4,5-dimethylthiazol-2-yl)-2,5-diphenyl tetrazolium bromide in the complete growth medium (MTT reagent; Sigma-Aldrich, St. Luis, MO, USA) was added to each well in a volume of 100 µL. The plates were incubated for three hours in the CO_2_ incubator under the abovementioned conditions. Subsequently, the MTT reagent was gently decanted, and the formed formazan crystals were dissolved in DMSO (Merck, Darmstadt, Germany). The absorbance was measured with an Infinite^®^ M200 spectrophotometer (Tecan Group Ltd., Mannedorf, Switzerland) at λ = 570 nm with a 690 nm reference wavelength.

#### 3.5.3. Evaluation of the Cell Numbers After Exposure to *bis*-Chalcone Derivatives with the Sulforhodamine B (SRB) Assay

After the treatment time specified in the experimental conditions, the cells were fixed with trichloroacetic acid (TCA; Sigma-Aldrich, St. Luis, MO, USA) at a final concentration of 12.5% and incubated for 1 h at 4 °C, followed by gentle rinsing with cold water and drying. Subsequently, a freshly prepared solution of 0.04% SRB (Sigma-Aldrich, St. Luis, MO, USA) in 1% acetic acid (Avantor Performance Materials Poland, Gliwice, Poland) was added, and plates were left at room temperature in the dark for 30 min. After this time, the plates were rinsed with 1% acetic acid. The protein-bound SRB was solubilized in 10 mM Tris base solution (BioShop, Burlington, ON, Canada), pH 10.5. The absorbance, proportional to the protein content, was measured using an Infinite^®^ M200 spectrophotometer (Tecan Group Ltd., Mannedorf, Switzerland) at λ = 520 nm.

#### 3.5.4. Statistical Analysis

Descriptive data are shown as the mean and standard deviation (SD). The data distribution was tested with the Shapiro–Wilk normality test, and Leven’s test was used to analyze the homogeneity of variances. One-way analysis of variance ANOVA was used for multiple comparison procedures, and the post hoc Tukey test was used to evaluate the differences between the control and study groups. Values with *p* < 0.05 were considered to be statistically significant. The data were analyzed using MS Excel 2016 (Microsoft Co., Redmond, WA, USA) and Statistica v.13.3 (Tibco Software Inc., Palo Alto, CA, USA).

## 4. Conclusions

In conclusion, a series of four *bis*-chalcone (**3a**–**3d**) have been synthesized using nonclassical conditions via MW- and MW-US-assisted Claisen–Schmidt condensation. The microwave methods align perfectly with the principles of green chemistry, as they prevent wastage and lower energy consumption. In the case of the synthesis of *bis*-chalcone systems, it was calculated that the use of green conditions supported by MW or MW-US factors led to an increase in the yield of the final products by 10–17%, with an approximately 570–600 times reduction in the reaction time over the classic method. The electrical energy consumption was estimated as a result of multiplying the power of the synthetic microwave device and the device’s operating time. As a result, due to the significant reduction in the response time, the total energy consumption has been reduced, and the overall environmental factor has improved. The use of MW and US for the synthesis of *bis*-chalcones significantly improves the process compared to the classical method. The cytotoxic and antiproliferative effects of the *bis*-chalcones against human MeWo and A375 melanoma cell lines were investigated via the colorimetric MTT and SRB assays. All the tested compounds had cytotoxic effects in the tested carcinoma cell lines. The present results showed high antiproliferative and cytotoxic potential, in particular for two selected *bis*-chalcone derivatives (**3b** and **3c**) on A375 and MeWo melanoma cells, especially at concentrations of 50 μM–200 μM at 24, 48 h, and 72 h of incubation (*p* < 0.05). The derivatives containing two hydroxy and two methoxy groups were the most effective against the cancer cells tested.

## Figures and Tables

**Table 1:** MW, MW-US, and classical methods comparison.

Compound	Classic Method			MW Method 100/200 W			MW-US Method 100 W–100 W		
	Temp [°C]	Time [h]	Yield [%]	Temp [°C]	Time [min.]	Yield [%]	Temp [°C]	Time [min.]	Yield [%]
**3a**	r.t. *	48	88	80–85	15/10	94/95	80–85	5	97
**3b**		48	79		15/10	87/90		5	92
**3c**		48	83		15/10	90/93		5	95
**3d**		48	85		15/10	92/93		5	96

* r.t. room temperature (~°C).

## Data Availability

Data are contained within this article or Supplementary Materials. Further inquiries can be directed to the corresponding author.

## Supplementary Materials

The following supporting information can be downloaded at: https://www.mdpi.com/article/10.3390/molecules29215171/s1, Table S1: The results of the MTT assay on the viability of MeWo cells incubated with chalcones 3a, 3b, 3c, 3d, ChO for 24 h, 48 h, or 72 h. Descriptive data were presented as mean ± SD. Results of ANOVA analysis and post hoc tests were calculated, including control (1) (0.2% DMSO-treated cells) as 100% of cell viability. Results of post hoc tests are written in a smaller font.; Table S2: The results of the SRB assay on the viability of MeWo cells incubated with chalcones 3a, 3b, 3c, 3d, ChO for 24 h, 48 h, or 72 h. Descriptive data were presented as mean ± SD. Results of ANOVA analysis and post hoc tests were calculated, including control (1) (0.2% DMSO-treated cells) as 100% of cell viability. Results of post hoc tests are written in a smaller font.; Table S3: The results of the MTT assay on the viability of A375 cells incubated with chalcones 3a, 3b, 3c, 3d, ChO for 24 h, 48 h, or 72 h. Descriptive data were presented as mean ± SD. Results of ANOVA analysis and post hoc tests were calculated, including control (1) (0.2% DMSO-treated cells) as 100% of cell viability. Results of post hoc tests are written in a smaller font.; Table S4: The results of the SRB assay on the viability of A375 cells incubated with chalcones 3a, 3b, 3c, 3d, ChO for 24 h, 48 h, or 72 h. Descriptive data were presented as mean ± SD. Results of ANOVA analysis and post hoc tests were calculated, including control (1) (0.2% DMSO-treated cells) as 100% of cell viability. Results of post hoc tests are written in a smaller font.
