# Supplementary material for: Synthesis of bis-Chalcones Based on Green Chemistry Strategies and Their Cytotoxicity Toward Human MeWo and A375 Melanoma Cell Lines
Source: Molecules. 2024 Oct 31;29(21):5171. doi: 10.3390/molecules29215171 (PMC11547983; doi:10.3390/molecules29215171)
Supplement: Supplementary file 1 [file molecules-29-05171-s001.zip › molecules-3230841-supplementary.pdf]

Synthesis of *bis*-Chalcones Based on Green Chemistry Strategies and Their Cytotoxicity Toward Human MeWo and A375 Melanoma Cell Lines

Dorota Olender [1,*], Anna Pawełczyk [1], Anna Leśków [2], Katarzyna Sowa-Kasprzak [1], Lucjusz Zaprutko [1] and Dorota Diakowska [2]

[1] Chair and Department of Organic Chemistry, Faculty of Pharmacy, Poznan University of Medical Sciences, Rokietnicka 3, 60-806 Poznań, Poland
[2] Department of Medical Biology, Wroclaw Medical University, Chalubinskiego 3, 50-368 Wrocław, Poland
* Correspondence: dolender@ump.edu.pl

Anticancer activity of bis-chalcone derivatives on human MeWo and A375 melanoma cell lines

Table S1. The results of the MTT assay on the viability of MeWo cells incubated with chalcones 3a, 3b, 3c, 3d, ChO for 24 h, 48 h, or 72 h. Descriptive data were presented as mean ± SD. Results of ANOVA analysis and post hoc tests were calculated, including control (1) (0.2% DMSO-treated cells) as 100% of cell viability. Results of post hoc tests are written in a smaller font.

Concentration	3a (%) (2)	3b (%) (3)	3c (%) (4)	3d (%) (5)	ChO (%) (6)	P-value
**24 h**						
10 µM	119.69 ± 25.17	129.61 ± 23.39	95.09 ± 33.24	96.26 ± 21.31	108.17 ± 24.76	0.025*<br>3 vs 4
25 µM	91.48 ± 21.66	119.57 ± 24.84	64.28 ± 27.16	74.50 ± 31.87	124.53 ± 8.28	<0.0001*<br>1 vs 4<br>2 vs 6<br>3 vs 4,5<br>4 vs 6<br>5 vs 6
50 µM	74.61 ± 15.87	42.99 ± 10.11	29.44 ± 8.87	81.10 ± 18.69	128.34 ± 22.23	<0.0001*<br>1 vs 2,3,4,6<br>2 vs 3,4,6<br>3 vs 5,6<br>4 vs 5,6<br>5 vs 6
100 µM	76.46 ± 15.05	14.13 ± 6.77	11.02 ± 4.02	79.95 ± 14.81	119.61 ± 16.91	<0.0001*<br>1 vs 2,3,4,5,6<br>2 vs 3,4,6<br>3 vs 5,6<br>4 vs 5,6<br>5 vs 6

150 µM	74.20<br>± 15.67	6.34<br>± 2.26	10.98<br>± 3.22	78.28<br>± 17.55	86.04<br>± 21.79	<0.0001*<br><br>1 vs 2,3,4,5<br>2 vs 3,4<br>3 vs 5,6<br>4 vs 5,6
200 µM	73.50<br>± 16.45	6.74<br>± 1.02	8.14<br>± 1.32	81.22<br>± 15.13	107.33<br>± 11.92	<0.0001*<br><br>1 vs 2,3,4,5<br>2 vs 3,4,6<br>3 vs 5,6<br>4 vs 5,6<br>5 vs 6
			**48h**			
10 µM	119.77<br>± 21.66	90.47<br>± 32.25	93.46<br>± 20.34	92.91<br>± 18.21	90.11<br>± 11.29	0.031*<br>2 vs 3,6
25 µM	103.85<br>± 17.98	69.82<br>± 39.52	66.37<br>± 25.32	99.21<br>± 21.70	90.11<br>± 21.18	0.006*<br>2 vs 4
50 µM	82.85<br>± 14.90	16.86<br>± 5.61	11.29<br>± 1.82	93.74<br>± 16.63	90.30<br>± 17.54	<0.0001*<br>1 vs 3,4<br>2 vs 3,4<br>3 vs 5,6<br>4 vs 5,6
100 µM	75.24<br>± 13.61	7.73<br>± 1.13	6.83<br>± 0.84	92.67<br>± 17.90	80.11<br>± 16.55	<0.0001*<br>1 vs 2,3,4,6<br>2 vs 3,4,5<br>3 vs 5,6<br>4 vs 5,6
150 µM	73.75<br>± 7.48	4.69<br>± 1.78	5.92<br>± 0.68	88.99<br>± 13.11	60.59<br>± 9.96	<0.0001*<br>1 vs 2,3,4,6<br>2 vs all<br>3 vs 5,6<br>4 vs 5,6<br>5 vs 6
200 µM	75.93<br>± 10.43	4.54<br>± 1.95	5.42<br>± 0.48	95.24<br>± 18.57	67.76<br>± 10.69	<0.0001*<br>1 vs 2,3,4,6<br>2 vs 3,4,5<br>3 vs 5,6<br>4 vs 5,6<br>5 vs 6
			**72h**			
10 µM	138.42<br>± 18.92	110.95<br>± 19.80	109.52<br>± 22.51	107.82<br>± 19.87	100.75<br>± 24.47	0.003*<br>1 vs 2<br>2 vs 4,5,6
25 µM	118.06<br>± 24.13	78.92<br>± 24.38	63.16<br>± 21.27	111.39<br>± 20.34	114.00<br>± 21.67	<0.0001*<br>1 vs 4<br>2 vs 3,4<br>3 vs 5,6<br>4 vs 5,6
50 µM	90.46<br>± 22.08	10.47<br>± 2.38	11.79<br>± 4.61	107.96<br>± 19.94	109.23<br>± 18.91	<0.0001*<br>1 vs 3,4<br>2 vs 3,4<br>3 vs 5,6<br>4 vs 5,6
100 µM	87.68	5.76	6.27	104.47	88.42	<0.0001*

	+ 21.89	+ 1.02	+ 0.36	+ 21.95	+ 9.23	1 vs 3,4<br>2 vs 3,4<br>3 vs 5,6<br>4 vs 5,6
150 µM	86.97<br>+ 13.64	5.02<br>+ 0.97	6.20<br>+ 0.25	106.32<br>+ 17.44	51.55<br>+ 16.18	<0.0001*<br>1 vs 3,4,6<br>2 vs 3,4,5,6<br>3 vs 5,6<br>4 vs 5,6<br>5 vs 6
200 µM	69.58<br>+ 13.89	5.01<br>+ 1.15	5.73<br>+ 0.45	98.38<br>+ 22.37	77.29<br>+ 13.09	<0.0001*<br>1 vs 2,3,4,6<br>2 vs 3,4,5<br>3 vs 5,6<br>4 vs 5,6<br>5 vs 6

*: statistically significant differences

Table S2. The results of the SRB assay on the viability of MeWo cells incubated with chalcones 3a, 3b, 3c, 3d, ChO for 24 h, 48 h, or 72 h. Descriptive data were presented as mean ± SD. Results of ANOVA analysis and post hoc tests were calculated, including control (1) (0.2% DMSO-treated cells) as 100% of cell viability. Results of post hoc tests are written in a smaller font.

Concentration	3a<br>(2)	3b<br>(3)	3c<br>(4)	3d<br>(5)	ChO<br>(6)	P-value
**24 h**						
10 µM	111.71<br>+ 26.97	103.49<br>+ 19.90	100.30<br>+ 22.53	112.71<br>+ 22.36	106.91<br>+ 22.12	0.667
25 µM	115.42<br>+ 27.18	97.56<br>+ 15.79	67.37<br>+ 21.62	110.95<br>+ 26.76	111.11<br>+ 22.41	<0.0001*<br>1 vs 4<br>2 vs 4<br>3 vs 4<br>4 vs 5,6
50 µM	116.62<br>+ 29.14	62.20<br>+ 20.12	37.29<br>+ 7.00	115.32<br>+ 25.36	117.07<br>+ 22.13	<0.0001*<br>1 vs 3,4<br>2 vs 3,4<br>3 vs 5,6<br>4 vs 5,6
100 µM	120.72<br>+ 35.06	41.64<br>+ 12.31	25.45<br>+ 5.71	117.59<br>+ 21.74	109.36<br>+ 21.62	<0.0001*<br>1 vs 3,4<br>2 vs 3,4<br>3 vs 5,6<br>4 vs 5,6
150 µM	116.50<br>+ 27.39	19.64<br>+ 1.14	25.45<br>+ 5.61	119.14<br>+ 23.49	84.58<br>+ 16.94	<0.0001*<br>1 vs 3,4<br>2 vs 3,4,6<br>3 vs 5,6<br>4 vs 5,6<br>5 vs 6
200 µM	115.29<br>+ 24.23	17.96<br>+ 1.02	20.06<br>+ 1.03	127.81<br>+ 24.71	94.69<br>+ 15.32	<0.0001*<br>1 vs 3,4,5<br>2 vs 3,4<br>3 vs 5,6<br>4 vs 5,6<br>5 vs 6

			**48h**			
10 µM	115.18<br>± 26.94	95.04<br>± 29.46	98.78<br>± 16.41	100.53<br>± 31.39	100.60<br>± 31.69	0.645
25 µM	112.89<br>± 34.77	67.97<br>± 26.88	66.21<br>± 23.92	110.51<br>± 25.32	118.14<br>± 30.17	<0.0001*<br>2 vs 3,4<br>3 vs 5,6<br>4 vs 5,6
50 µM	111.13<br>± 26.08	32.07<br>± 9.04	22.56<br>± 1.98	113.09<br>± 16.52	115.91<br>± 31.41	<0.0001*<br>1 vs 3,4<br>2 vs 3,4<br>3 vs 5,6<br>4 vs 5,6
100 µM	110.68<br>± 23.70	14.56<br>± 1.44	14.22<br>± 1.03	117.32<br>± 9.02	103.67<br>± 20.77	<0.0001*<br>1 vs 3,4<br>2 vs 3,4<br>3 vs 5,6<br>4 vs 5,6
150 µM	109.86<br>± 25.98	13.11<br>± 0.38	14.17<br>± 1.03	118.21<br>± 24.46	74.65<br>± 29.52	<0.0001*<br>1 vs 3,4<br>2 vs 3,4,6<br>3 vs 5,6<br>4 vs 5,6<br>5 vs 6
200 µM	105.65<br>± 23.49	13.31<br>± 0.71	13.78<br>± 0.63	120.21<br>± 20.93	94.53<br>± 23.85	<0.0001*<br>1 vs 3,4<br>2 vs 3,4<br>3 vs 5,6<br>4 vs 5,6<br>5 vs 6
			**72h**			
10 µM	110.31<br>± 23.83	94.71<br>± 33.88	118.49<br>± 22.08	119.19<br>± 31.60	109.60<br>± 24.55	0.254
25 µM	107.74<br>± 24.23	60.11<br>± 17.75	68.35<br>± 25.66	121.21<br>± 25.25	122.58<br>± 21.35	<0.0001*<br>1 vs 3,4<br>2 vs 3,4<br>3 vs 5,6<br>4 vs 5,6
50 µM	104.80<br>± 24.00	11.99<br>± 1.48	14.25<br>± 1.17	121.97<br>± 20.77	119.73<br>± 22.53	<0.0001*<br>1 vs 3,4<br>2 vs 3,4<br>3 vs 5,6<br>4 vs 5,6
100 µM	97.07<br>± 19.50	11.01<br>± 0.71	10.85<br>± 0.46	118.65<br>± 21.46	104.08<br>± 7.37	<0.0001*<br>1 vs 3,4,5<br>2 vs 3,4,5<br>3 vs 5,6<br>4 vs 5,6
150 µM	98.49<br>± 20.95	10.91<br>± 0.51	11.23<br>± 0.77	122.74<br>± 21.16	80.81<br>± 17.30	<0.0001*<br>1 vs 3,4,5<br>2 vs 3,4,5<br>3 vs 5,6<br>4 vs 5,6<br>5 vs 6
200 µM	98.24<br>± 23.06	11.00<br>± 0.57	11.60<br>± 0.84	127.19<br>± 22.21	81.50<br>± 14.53	<0.0001*<br>1 vs 3,4,5<br>2 vs 3,4,5<br>3 vs 5,6

						4 vs 5,6<br>5 vs 6

Table S3. The results of the MTT assay on the viability of A375 cells incubated with chalcones 3a, 3b, 3c, 3d, ChO for 24 h, 48 h, or 72 h. Descriptive data were presented as mean ± SD. Results of ANOVA analysis and post hoc tests were calculated, including control (1) (0.2% DMSO-treated cells) as 100% of cell viability. Results of post hoc tests are written in a smaller font.

Concentration	3a<br>(2)	3b<br>(3)	3c<br>(4)	3d<br>(5)	ChO<br>(6)	P-value
**24 h**						
10 µM	88.63<br>± 33.86	79.45<br>± 23.00	52.70<br>± 27.60	90.33<br>± 34.01	101.85<br>± 31.61	0.005*<br>1 vs 4<br>4 vs 6
25 µM	37.97<br>± 16.28	65.33<br>± 29.91	18.94<br>± 9.71	58.92<br>± 27.22	131.52<br>± 44.74	<0.0001*<br>1 vs 2,4,5<br>2 vs 6<br>3 vs 4,6<br>4 vs 5,6<br>5 vs 6
50 µM	26.58<br>± 10.52	8.72<br>± 3.21	10.93<br>± 4.55	40.91<br>± 23.96	127.72<br>± 22.59	<0.0001*<br>1 vs all<br>2 vs 6<br>3 vs 5,6<br>4 vs 5,6<br>5 vs 6
100 µM	27.19<br>± 10.69	10.47<br>± 5.72	8.58<br>± 4.82	36.45<br>± 15.05	130.31<br>± 48.30	<0.0001*<br>1 vs all<br>2 vs 6<br>3 vs 6<br>4 vs 6<br>5 vs 6
150 µM	19.29<br>± 5.08	8.40<br>± 8.13	5.45<br>± 3.45	28.19<br>± 13.32	115.55<br>± 35.55	<0.0001*<br>1 vs 2,3,4,5<br>2 vs6<br>3 vs 6<br>4 vs 5,6<br>5 vs 6
200 µM	20.51<br>± 6.30	4.79<br>± 3.00	4.33<br>± 2.16	25.53<br>± 12.30	127.88<br>± 41.26	<0.0001*<br>1 vs all<br>2 vs 6<br>3 vs 6<br>4 vs 6<br>5 vs 6
**48h**						
10 µM	72.70<br>± 19.65	54.36<br>± 13.86	61.78<br>± 24.54	72.52<br>± 29.74	103.98<br>± 22.60	<0.0001*<br>1 vs 3,4<br>2 vs 6<br>3 vs 6<br>4 vs 6<br>5 vs 6
25 µM	50.12<br>± 10.79	16.41<br>± 14.76	12.80<br>± 4.33	76.47<br>± 21.33	110.27<br>± 23.80	<0.0001*<br>1 vs 2,3,4,5<br>2 vs all<br>3 vs 5,6

						4 vs 5,6<br>5 vs 6
50 µM	35.03<br>± 8.13	2.00<br>± 0.27	2.29<br>± 0.24	74.71<br>± 16.17	96.46<br>± 21.97	<0.0001*<br>1 vs 2,3,4,5<br>2 vs all<br>3 vs 5,6<br>4 vs 5,6<br>5 vs 6
100 µM	33.54<br>± 6.97	1.74<br>± 0.30	1.80<br>± 0.21	67.27<br>± 17.48	108.62<br>± 20.95	<0.0001*<br>1 vs 2,3,4,5<br>2 vs all<br>3 vs 5,6<br>4 vs 5,6<br>5 vs 6
150 µM	29.56<br>± 8.72	1.67<br>± 0.30	1.88<br>± 0.22	72.79<br>± 13.27	73.49<br>± 21.54	<0.0001*<br>1 vs all<br>2 vs all<br>3 vs 5,6<br>4 vs 5,6
200 µM	28.20<br>± 6.44	1.65<br>± 0.67	1.88<br>± 0.20	69.07<br>± 14.45	85.18<br>± 23.57	<0.0001*<br>1 vs 2,3,4,5<br>2 vs all<br>3 vs 5,6<br>4 vs 5,6
**72h**						
10 µM	53.95<br>± 24.08	71.97<br>± 14.90	91.30<br>± 32.34	114.94<br>± 28.73	112.93<br>± 36.38	<0.0001*<br>1 vs 2<br>2 vs 4,5,6<br>3 vs 5,6
25 µM	41.04<br>± 16.82	5.69<br>± 1.03	22.26<br>± 3.67	98.47<br>± 16.11	106.72<br>± 32.67	<0.0001*<br>1 vs 2,3,4<br>2 vs 3,5,6<br>3 vs 5,6<br>4 vs 5,6
50 µM	39.23<br>± 10.46	2.52<br>± 0.56	3.30<br>± 0.42	100.63<br>± 18.17	120.57<br>± 28.30	<0.0001*<br>1 vs 2,3,4,6<br>2 vs all<br>3 vs 5,6<br>4 vs 5,6
100 µM	34.14<br>± 9.00	2.38<br>± 0.64	2.55<br>± 0.39	94.55<br>± 19.20	109.93<br>± 19.38	<0.0001*<br>1 vs 2,3,4<br>2 vs all<br>3 vs 5,6<br>4 vs 5,6
150 µM	30.84<br>± 8.21	2.41<br>± 0.45	2.49<br>± 0.31	96.39<br>± 18.47	89.89<br>± 17.71	<0.0001*<br>1 vs 2,3,4<br>2 vs all<br>3 vs 5,6<br>4 vs 5,6
200 µM	28.85<br>± 9.10	2.29<br>± 0.96	2.69<br>± 0.37	92.24<br>± 21.14	82.01<br>± 20.51	<0.0001*<br>1 vs 2,3,4,6<br>2 vs all<br>3 vs 5,6<br>4 vs 5,6

Table S4. The results of the SRB assay on the viability of A375 cells incubated with chalcones 3a, 3b, 3c, 3d, ChO for 24 h, 48 h, or 72 h. Descriptive data were presented as mean ± SD. Results of ANOVA analysis and post hoc tests were calculated, including control (1) (0.2% DMSO-treated cells) as 100% of cell viability. Results of post hoc tests are written in a smaller font.

Concentration	3a<br>(2)	3b<br>(3)	3c<br>(4)	3d<br>(5)	ChO<br>(6)	P-value
**24 h**						
10 µM	108.08<br>± 18.21	79.81<br>± 10.43	85.99<br>± 10.00	103.84<br>± 10.32	85.28<br>± 20.33	<0.0001*<br>1 vs 3<br>2 vs 3,4,6<br>3 vs 5
25 µM	89.64<br>± 13.13	58.21<br>± 15.40	29.63<br>± 3.62	109.16<br>± 11.14	94.74<br>± 14.06	<0.0001*<br>1 vs 3,4<br>2 vs 3,4,5<br>3 vs all<br>4 vs 5,6
50 µM	83.02<br>± 11.72	24.55<br>± 1.15	23.89<br>± 1.06	105.47<br>± 11.47	95.19<br>± 16.23	<0.0001*<br>1 vs 2,3,4<br>2 vs 3,4,5<br>3 vs 5,6<br>4 vs 5,6
100 µM	79.62<br>± 15.66	21.39<br>± 1.09	22.16<br>± 1.30	101.05<br>± 6.26	101.87<br>± 18.97	<0.0001*<br>1 vs 2,3,4<br>2 vs all<br>3 vs 5,6<br>4 vs 5,6
150 µM	73.53<br>± 13.86	21.58<br>± 0.74	23.46<br>± 1.18	103.15<br>± 7.48	69.11<br>± 26.20	<0.0001*<br>1 vs 2,3,4,6<br>2 vs 3,4,5<br>3 vs 5,6<br>4 vs 5,6<br>5 vs 6
200 µM	74.30<br>± 13.04	22.33<br>± 1.40	25.60<br>± 3.65	104.83<br>± 8.09	83.95<br>± 12.63	<0.0001*<br>1 vs 2,3,4,6<br>2 vs 3,4,5<br>3 vs 5,6<br>4 vs 5,6<br>5 vs 6
**48h**						
10 µM	86.64<br>± 20.05	47.54<br>± 7.55	77.05<br>± 12.61	94.15<br>± 14.85	90.43<br>± 30.30	<0.0001*<br>1 vs 3<br>2 vs 3<br>3 vs all<br>4 vs 3<br>5 vs 3<br>6 vs 3
25 µM	76.40<br>± 12.23	15.42<br>± 8.07	20.95<br>± 5.24	86.83<br>± 10.79	109.28<br>± 28.85	<0.0001*<br>1 vs 2,3,4<br>2 vs 3,4,6<br>3 vs 5,6<br>4 vs 5,6<br>5 vs 6
50 µM	64.59<br>± 10.83	7.49<br>± 0.29	7.84<br>± 1.00	92.63<br>± 15.14	102.60<br>± 29.87	<0.0001*<br>1 vs 2,3,4<br>2 vs all<br>3 vs 5,6

						4 vs 5,6
100 µM	57.64<br>+ 12.90	7.31<br>+ 0.56	7.59<br>+ 0.83	92.62<br>+ 9.54	103.29<br>+ 18.70	<0.0001*<br>1 vs 2,3,4<br>2 vs all<br>3 vs 5,6<br>4 vs 5,6
150 µM	56.72<br>+ 12.64	7.42<br>+ 0.62	7.32<br>+ 0.39	95.60<br>+ 11.77	82.14<br>+ 26.75	<0.0001*<br>1 vs 2,3,4<br>2 vs all<br>3 vs 5,6<br>4 vs 5,6
200 µM	46.09<br>+ 9.38	7.37<br>+ 0.67	7.52<br>+ 0.46	87.20<br>+ 10.91	90.55<br>+ 20.63	<0.0001*<br>1 vs 2,3,4<br>2 vs all<br>3 vs 5,6<br>4 vs 5,6
**72h**						
10 µM	63.36<br>+ 44.45	76.99<br>+ 24.67	146.54<br>+ 58.47	133.07<br>+ 41.26	102.31<br>+ 48.61	<0.0001*<br>2 vs 4,5<br>3 vs 4
25 µM	58.02<br>+ 41.16	9.15<br>+ 0.58	35.85<br>+ 19.17	136.40<br>+ 43.18	132.74<br>+ 52.27	<0.0001*<br>1 vs 3,4<br>2 vs 3,5,6<br>3 vs 5,6<br>4 vs 5,6
50 µM	55.63<br>+ 34.85	7.20<br>+ 1.74	6.89<br>+ 0.37	136.16<br>+ 42.10	144.65<br>+ 60.37	<0.0001*<br>1 vs 3,4<br>2 vs 3,4,5,6<br>3 vs 5,6<br>4 vs 5,6
100 µM	55.21<br>+ 24.77	6.54<br>+ 0.40	6.77<br>+ 0.42	131.88<br>+ 42.14	151.85<br>+ 54.92	<0.0001*<br>1 vs 2,3,4,6<br>2 vs all<br>3 vs 5,6<br>4 vs 5,6
150 µM	46.54<br>+ 21.14	6.57<br>+ 0.35	6.54<br>+ 0.28	132.81<br>+ 38.05	108.45<br>+ 38.12	<0.0001*<br>1 vs 2,3,4<br>2 vs all<br>3 vs 5,6<br>4 vs 5,6
200 µM	40.21<br>+ 22.44	6.68<br>+ 0.86	7.20<br>+ 1.00	124.94<br>+ 38.06	119.60<br>+ 42.78	<0.0001*<br>1 vs 2,3,4<br>2 vs 5,6<br>3 vs 5,6<br>4 vs 5,6
